# The Duties of Royalty

**Published:** 1907-04-27

**Authors:** 


### The Hospital

A Journal of

The Medical Sciences and Hospital Administration.

New Series. No. 4, Vol. I. [No. 1076, Vol. XLII.] SATURDAY, APRIL 27, 1907.

### THE DUTIES OF ROYALTY.

Medical Institutions, and especially Hospitals, in this country in the present day owe more to the Royal Family of England than it would be easy to express in words. Her Majesty, the late Queen Victoria, although the fact was not generally recognised, took the keenest interest in the suffering and the sick, and in all institutions for their benefit. We can go back nearly forty years to the day, when we first had occasion to realise Queen Victoria's interest in the hospitals. Her late Majesty used to watch the progress of various institutions for the sick, and she would spontaneously take occasion to intimate her satisfaction, where she considered the work had been exceptionally well done. On one such occasion she caused a letter to be written, in support of a hospital of international interest, which was so emphatic that the late John Delane, as Editor of the *Times*, thought it prudent to make direct inquiries as to its authenticity, before its publication. No doubt, this keen interest in the welfare of the sick is one great reason why Queen Victoria will ever be beloved as the Mother of her people.

King Edward VII. when Prince of Wales, as the history of his public life proves, never hesitated to render personal service in the cause of the sick. During the last twenty years few men have done as much as His Majesty, by personal service, in this direction. The establishment of King Edward's Hospital Fund for London and of the League of Mercy, the services he rendered to Guy's Hospital, and the success which has attended these movements, owing largely to the King's personal interest in, and work for the sick, will go down, through the ages, as some of the brightest and best work which a monarch has ever done.

Glasgow during the present week has been *en fête*, and during seven hours of three working days, the Prince and Princess of Wales have given themselves up to ceremonial, social and public functions, to an extent which is marvellous from the volume of energy expended and remarkable for the spirit which underlies the devotion to duty which it represents. As President of the King's Fund and of the League of Mercy, the Prince of Wales gladly devotes a definite amount of time, and all his energy and attention at such periods, each year, to work for the sick. The Princess of Wales has identified herself to a large extent with the children, and she yields to no one in her devotion to the public duties which devolve upon her from time to time. We have no doubt that the Medical School of Glasgow will greatly benefit by the visit of the Prince of Wales to its University, on Tuesday, and by the interest he displayed in its work and welfare. We can well believe, however, that the Prince felt an especial interest in Wednesday's proceedings, because he then laid the foundation-stone of the Royal Infirmary, situate in the Gatehouse Block, which marks a new departure in hospital construction, and will probably constitute a new unit. The Glasgow Royal Infirmary, if erected from the accepted plans as they stand to-day, despite the limitations of its site, and the difficulties which have had to be overcome, will certainly constitute one of the most interesting of modern hospitals. Unfortunately, some eight years must elapse, before the whole of the reconstruction, now commenced, can be carried out in its entirety. We hope the wiser heads amongst the Managers, and the general sentiments of the architectural profession, backed by the just pride of the citizens of Glasgow, will prevent, during these eight years, any fussy interference, or ill-considered tinkering that may mar the excellence of the plans, as they stand to-day.

The Prince of Wales is an able critic, and a keen inspector of hospitals and their work. Not only is it an honour, but a very real help to trained administrators in charge of a great hospital, that such an institution should be visited by His Royal Highness. Like his parents, the Prince has a trained eye for efficiency, and wherever he goes, this most useful faculty is apt to leave its mark in results. We hope the inhabitants of Glasgow and the people of Scotland will appreciate the generosity in hard work which their Royal Highnesses displayed, during their recent visit. We believe that this visit will be memorable in the history of such events, from the fact, that never before has any city received such a volume of personal service from Royalty, as that rendered to the citizens, institutions, and interests of Glasgow, by the Prince and Princess of Wales, during the current week.

